# Left versus right carotid artery IMT: differential impact of age, gender, and cardiovascular risk factors

**DOI:** 10.1007/s10554-024-03245-1

**Published:** 2024-09-26

**Authors:** Belinda Stevens, Talib Abdool-Carrim, Angela J. Woodiwiss

**Affiliations:** Cardiovascular Pathophysiology and Genomics Research Unit, School of Physiology, Faculty of Health Sciences, University of the Witwatersrand Medical School, 7 York Road, Parktown, Johannesburg, 2193 South Africa

**Keywords:** Carotid IMT, Body side, Age, Gender, Cardiovascular risk factors

## Abstract

Carotid artery intima-media thickness (IMT), an important clinical marker of atherosclerosis, is used widely in screening for cardiovascular risk and prognosis. Measurements of carotid artery IMT are made on both the left and right sides of the body, however as per the Mannheim consensus, an average of these measurements is usually reported. Nevertheless, there is considerable debate whether there are side differences in the carotid artery IMT in terms of both measurements and determinants. In a large sample of Caucasian patients (n = 1888) referred for cardiovascular risk assessment, we compared the left and the right common carotid artery IMT measurements, and assessed whether age, gender and cardiovascular risk factors have differential effects. We found that the left common carotid artery IMT (0.7141 ± 0.1733 mm) is larger than the right (0.6861 ± 0.1594, p < 0.0001), but not in the young (< 30 years) or the elderly (> 69 years), and that this side difference is less in women (0.019 ± 0.116 mm) than in men (0.036 ± 0.148 mm, p < 0.001). In addition to age (p < 0.0001) and gender (p < 0.0001), the left common carotid artery IMT was determined by dyslipidaemia (protective, p = 0.016) and diabetes mellitus (p = 0.022); whereas the right common carotid artery IMT was determined by hypertension (p = 0.0002). The differential determinants of left versus right common carotid artery IMT were similar in men and women, and in young and old. In conclusion, side differences in measurements of the common carotid artery IMT depend upon age and gender. In addition, cardiovascular risk factors have differential effects on the left and right common carotid artery IMT.

**Supplementary Information:**

The online version contains supplementary material available at 10.1007/s10554-024-03245-1.

## Introduction

Carotid artery intima-media thickness (IMT) is an important clinical marker of atherosclerosis and hence is used widely in screening for cardiovascular risk [1–5]. In addition, changes in carotid artery IMT over time have prognostic utility [6, 7]. Although some studies indicate that the left carotid artery IMT is larger than the right [8–16], other studies report no differences between the left and right carotid artery IMT [11, 13, 17–21]. There is therefore still considerable debate as to whether these side differences occur, and consequently, as per the Mannheim consensus, an average of the left and right carotid IMT is reported [22].

It has been suggested that whether differences between the left versus the right carotid artery IMT occur may depend on age, and the presence of cardiovascular disease. However, these data are inconsistent. A greater left versus right carotid artery IMT has been reported in healthy individuals aged between 35 and 65 years, with no differences reported in those less than 35 years or greater than 65 years of age [9]. In contrast, a study in youth (mean age 14.7 years) reported a greater IMT in the left compared to the right common carotid artery [23]. Regarding the presence of cardiovascular disease, some studies report a larger left versus right carotid artery IMT in persons with cardiovascular disease [8, 11–13, 15], with no side differences reported in healthy individuals [11, 13, 17–20]. Nevertheless, in contrast, a greater left versus right carotid artery IMT has been reported in the absence of cardiovascular disease [8–10, 15, 16], with no side differences reported in those with cardiovascular disease [11, 17, 21]. Furthermore, despite carotid artery IMT being greater in men compared to women [8, 24–27], and increasing with age [1, 4, 5], only one study has previously reported an interactive effect of age and gender on side differences in carotid artery IMT [14].

Although, numerous studies show that a number of cardiovascular risk factors (namely hypertension, dyslipidaemia and diabetes) determine carotid artery IMT, there is limited data as to whether these risk factors similarly affect the left and the right carotid artery IMT. To our knowledge only one study has previously assessed potential differential impacts of cardiovascular risk factors on the left versus the right carotid artery IMT. This study, in an Asian population, reported that the right carotid artery IMT correlated better with haemodynamic parameters compared to the left carotid artery IMT which showed better correlations with biochemical indices [9]. Further data are therefore required to determine whether cardiovascular risk factors have a differential impact on the left and right carotid artery IMT in other populations, and in men versus women.

The presence of side differences in carotid IMT and the potential differential impact of age, gender and cardiovascular risk factors is not trivial. The common practise of reporting an average of the left and right carotid artery IMT, as per the Mannheim consensus [22], may result in important information being overlooked if side differences do occur. Indeed, in a prospective study of over 7400 individuals, in those with increased carotid IMT on either the left or the right side, only 24% had increased carotid IMT on both the left and right side [28]. Hence, over 75% had an increased carotid IMT on only one side. Consequently, the reporting of an average of left and right carotid IMT is likely to overlook a number of individuals who may have increased carotid IMT. Furthermore, if the impact of age, gender and cardiovascular risk factors differs between the left and the right carotid artery IMT, then there may be subgroups where reporting on the left and right carotid artery IMT as opposed to the average is particularly pertinent. We therefore aimed to firstly determine in a large Caucasian population whether there is a difference between the left and right common carotid artery IMT. Secondly, we aimed to determine whether age, gender and cardiovascular risk factors have a differential impact on the left compared to the right common carotid artery IMT in this population.

## Methods

A retrospective study was undertaken to determine whether there were differences in left versus right carotid IMT, and the impacts of age, gender and cardiovascular risk factors on any differences observed. The study was approved by the University of the Witwatersrand Human Research Ethics Committee and the private hospital. The ethics clearance number is M16-03-14 renewed as M21-06-94.

### Participants

A retrospective record review of patient hospital files in the Vascular Unit at a private hospital in Johannesburg, Gauteng, South Africa, was performed. The data pertaining to carotid artery ultrasonic evaluation performed on patients referred for cardiovascular risk for the time period between 1 January 1999 and 31 December 2015, was extracted. Carotid artery ultrasonic evaluation was performed on these patients at the request of their managing physicians. For each patient, the data for only the initial evaluation was extracted. A total of 1934 records were extracted. The majority (97.6%) of the records were from Caucasian patients, the other race groups were inadequately represented. As the samples sizes were too small for each of the other race groups, for the purposes of this study only the data extracted for Caucasian patients (n = 1888) was analysed.

### General characteristics and cardiovascular risk factors

Age, gender and race group were extracted from each patient’s hospital file. Hypertension was defined as systolic blood pressure ≥ 140 mmHg and/or a diastolic blood pressure ≥ 90 mmHg and/or the use of antihypertensive medications. Dyslipidaemia was defined as a total cholesterol > 5.5 mmmol/l and/or the use of lipid lowering medication (statins). Diabetes mellitus (type I or II) was defined as a fasting glucose > 7.0 mmol/l and/or and HbA_1c_ > 7.0 mmol/l.

### Common carotid artery IMT

Carotid IMT was determined by the same investigator (BS) using high resolution B-mode ultrasound employing a linear array 7.5 MHz probe as recommended [22]. Three images of at least 1 cm length of the far wall of the distal portion of both the right and the left common carotid artery from an optimal angle of incidence (defined as the longitudinal angle of approach where both branches of the internal and external carotid artery are visualized simultaneously) over a selected length 10 mm from the bifurcation were obtained. Intima-media thickness was defined as the distance between the leading edge of the lumen-intima interface and the leading edge of the media-adventitia interface [22]. Carotid IMT measurements were determined manually with three images of 3 measurements within a 10 mm length taken per side (in keeping with guidelines at the time) up until semi-automated border-detection software (Edgeware) became available. With the use of semi-automated border-detection software, a selected distance along the common carotid artery of no less than 10 mm yielded a number of individual points of assessment providing a minimum, maximum and mean measure. The machines used were the Toshiba ECOCEE (Toshiba Corporation, Minato, Tokyo, Japan), and Hewlett Packard Sonos (Hewlett-Packard Medical Products Group, USA) up until 2008, followed by the Philips HD11 (Philips, Amsterdam, Netherlands) until 2014 and thereafter the GE Logiq (GE HealthCare, USA).

### Data analysis

Database management and statistical analyses were performed with SAS software, version 9.4 (The SAS Institute, Cary, NC). Continuous variables are expressed as mean (± SD). Dichotomous variables are expressed as percentages. Side specific median and 75th percentile thresholds for common carotid artery IMT were derived from normotensive, non-diabetic, and non-dyslipidaemic patients from the study sample (n = 439, 56.3% men). In addition, age, gender and side specific thresholds as defined in the ARIC study [4], as well as a common (non-side specific) threshold of 0.68 as defined by Sun et al. [10], were employed. The left and right common carotid artery IMT measurements were compared using unpaired Student’s t test and Bland–Altman analysis. Bivariate and multivariate regression analyses were performed to assess the independent determinants of left versus right common carotid artery IMT. Multiple logistic regression analysis was performed to assess the determinants of increased common carotid artery IMT using the various thresholds defined above. In order to assess the potential impact of gender, all analyses were performed separately in women and men. The determinants of common carotid IMT were also assessed separately in young (age < 50 years in men and age < 55 years in women) and old (age ≥ 50 years in men and age ≥ 55 years in women).

## Results

### Participant characteristics

Table 1 shows the general characteristics of the study sample. A similar number of men and women were screened. A high proportion had dyslipidaemia, however only modest proportions had either hypertension or diabetes mellitus. In accordance with current therapeutic practice, all patients with dyslipidaemia were receiving lipid lowering medication (statins). The men and women were of similar age, however a greater proportion of women had dyslipidaemia, whereas a greater proportion of men had hypertension or diabetes mellitus (Table 1). Most of the patients (58%) had a combination of 2 risk factors (Table 1), which were predominantly a combination of dyslipidaemia and hypertension (78%). These proportions were similar in women and men (Table 1). Both the left and the right common carotid artery IMT were greater in the men than in the women (Table 1).

**Table 1:** Characteristics of the study sample, and the women and men separately

	All patients	Women	Men
n	1888	889	999
% male (n)	52.91 (999)	–	–
Age (years)	54.85 ± 12.03	55.63 ± 12.31	54.15 ± 11.74
% Hypertension (n)	17.27 (326)	13.39 (119)	20.72 (207)**
% Dyslipidaemia (n)	74.52 (1407)	77.50 (689)	71.87 (718)*
% Diabetes mellitus (n)	10.22 (193)	5.85 (52)	14.11 (141)**
% Risk factors^†^ (1/2/3)	58.21/11.81/6.73	64.23/10.01/4.16	52.85/13.41/9.01
Left CCA IMT (mm)	0.714 ± 0.173	0.684 ± 0.147	0.741 ± 0.190**
Min	0.313	0.324	0.313
Max	2.120	1.510	2.120
Right CCA IMT (mm)	0.686 ± 0.159	0.666 ± 0.140	0.704 ± 0.173**
Min	0.244	0.244	0.394
Max	1.940	1.706	1.940
Average CCA IMT (mm)	0.700 ± 0.152	0.675 ± 0.131	0.723 ± 0.166**
Min	0.284	0.284	0.418
Max	1.940	1.329	1.940
Left–right CCA IMT diff. (mm)	0.028 ± 0.134	0.019 ± 0.116	0.036 ± 0.148*

*CCA* common carotid artery, *IMT* intima media thickness, *average* average of left and right CCA IMT, *diff.* difference

*p < 0.001, **p < 0.0001 for comparisons between men and women

^†^Proportion of patients with 1, 2 or 3 of risk factors (hypertension, diabetes mellitus, dyslipidaemia) in combination

### Comparisons of left and right common carotid artery IMT

**Fig. 1:**
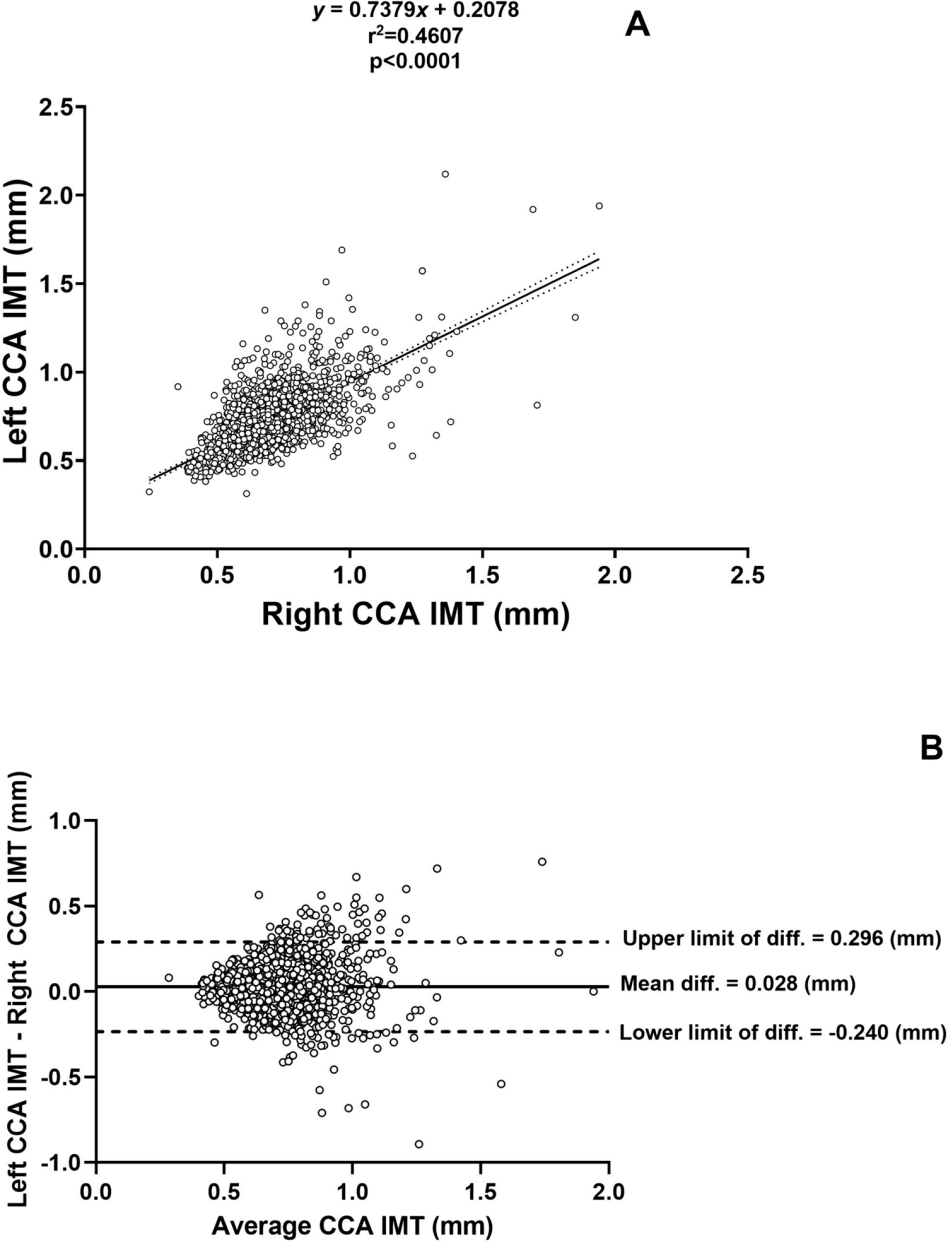
Correlation of left versus right common carotid IMT in the study sample (**A**), and Bland–Altman plot of left versus right common carotid IMT in the study sample (**B**)

Figure 1 shows the correlation between the left and right common carotid artery IMT measurements (panel A) and the Bland–Altman analysis to identify potential differences between the left and right common carotid artery IMT measurements (panel B). As expected the left and right common carotid artery IMT measurements were correlated (Fig. 1A), however the left common carotid artery IMT was larger than the right common carotid artery IMT with a mean difference of 0.028 mm (Fig. 1B and Table 1).

**Fig. 2:**
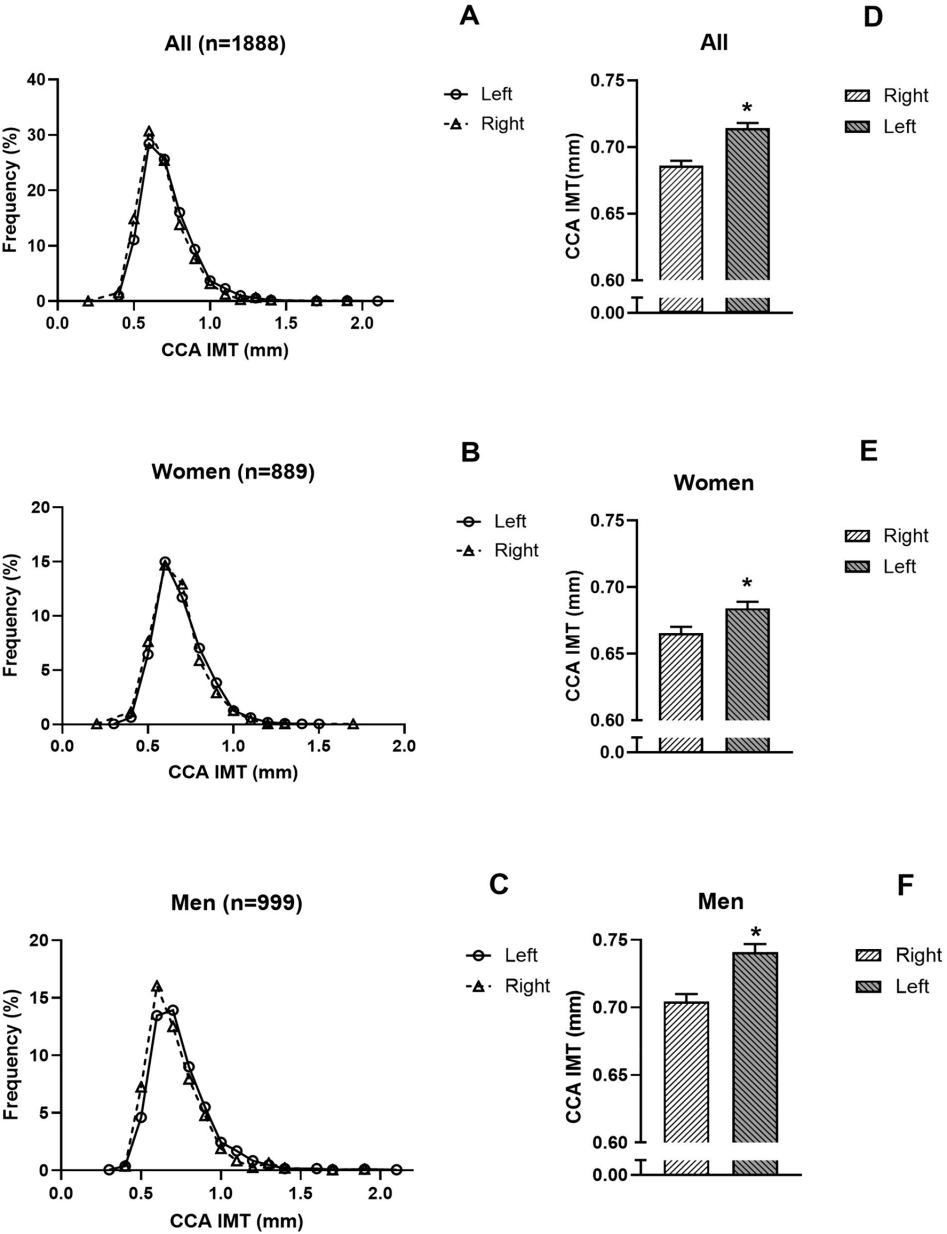
Frequency distribution of left and right common carotid IMT in the study sample (**A**), and in women (**B**) and men (**C**) separately, and comparisons of mean left and right common carotid IMT in the study sample (**D**), and in women (**E**) and men (**F**) separately. *p < 0.0001 for comparison of left versus right CCA IMT

Figure 2 shows the comparisons of the left and right common carotid artery IMT in the study sample (panels A and D), and in the women (panels B and E) and the men (panels C and F) separately. The left common carotid artery IMT was consistently greater than the right common carotid artery IMT. However, the difference in the common carotid artery IMT between the left and right sides was greater in the men than in the women (Table 1).

### Impact of age and gender on comparisons between left and right common carotid artery IMT

**Fig. 3:**
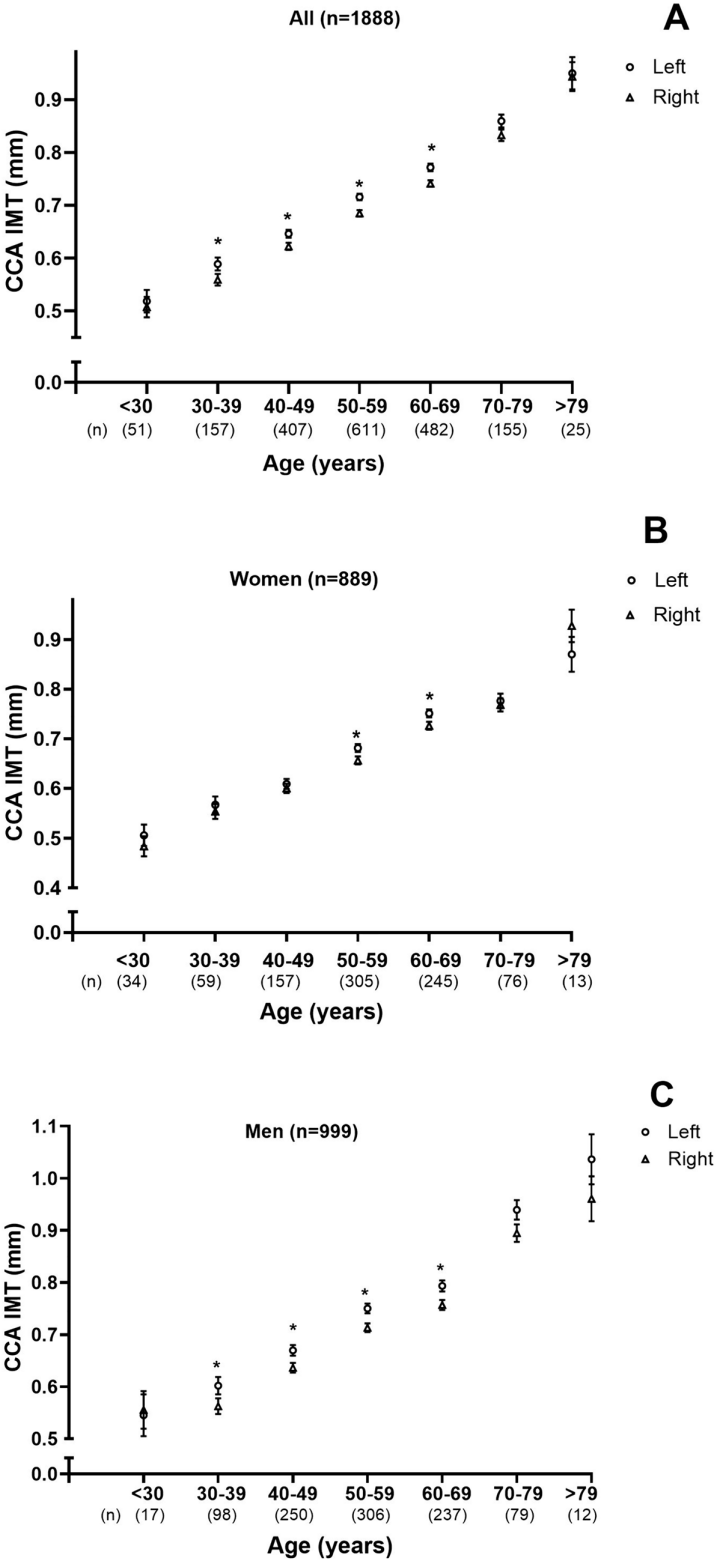
Comparison of left versus right common carotid IMT at each decade of age in the study sample (**A**), and in women (**B**) and men (**C**) separately. CCA IMT, common carotid intima media thickness; *p < 0.0001 for comparison of left versus right CCA IMT

Both the left and the right common carotid artery IMT increased per decade of age (Fig. 3). The relationship between common carotid artery IMT and age was noted in all patients (Fig. 3A, Tables S1, 2), and in both women (Fig. 3B, Tables S1, 2) and men (Fig. 3C, Tables S1 and 2). Despite the strong relationships between both left and right common carotid artery IMT and age (Tables S1, 2), the differences between the left and the right common carotid artery IMT measurements were not noted across all decades of age. In all patients (Fig. 3A) and in men (Fig. 3C), these differences were noted from age 30 to 69 years, whereas in women (Fig. 3B) these differences were only noted from age 50 to 69 years. No differences were noted between the left and the right common carotid artery IMT measurements in patients younger than 30 years or older than 69 years (Fig. 3).

**Table 2:** Determinants of left and right common carotid artery IMT in multivariate models

Determinant	Partial r (95% CI)	Std. beta ± sem	p value
All patients—left common carotid artery IMT			
Age	0.479 (0.443 to 0.513)	0.476 ± 0.020	< 0.0001
Gender (male)	0.202 (0.158 to 0.245)	0.179 ± 0.020	< 0.0001
Hypertension	0.029 (− 0.016 to 0.074)	0.029 ± 0.023	0.203
Dyslipidaemia	− 0.056 (− 0.101 to − 0.011)	− 0.049 ± 0.020	0.016
Diabetes mellitus	0.053 (0.008 to 0.098)	0.052 ± 0.022	0.022
All patients—right common carotid artery IMT			
Age	0.500 (0.465 to 0.533)	0.498 ± 0.020	< 0.0001
Gender (male)	0.163 (0.118 to 0.206)	0.142 ± 0.020	< 0.0001
Hypertension	0.087 (0.042 to 0.132)	0.085 ± 0.023	0.0002
Dyslipidaemia	− 0.055 (− 0.100 to − 0.010)	− 0.048 ± 0.020	0.017
Diabetes mellitus	− 0.009 (− 0.055 to 0.036)	− 0.009 ± 0.022	0.681
Women only—left common carotid artery IMT			
Age	0.502 (0.450 to 0.549)	0.500 ± 0.029	< 0.0001
Hypertension	0.080 (0.015 to 0.146)	0.079 ± 0.033	0.017
Dyslipidaemia	− 0.072 (− 0.137 to − 0.006)	− 0.062 ± 0.029	0.033
Diabetes mellitus	0.004 (− 0.062 to 0.070)	0.004 ± 0.032	0.909
Women only—right common carotid artery IMT			
Age	0.528 (0.479 to 0.574)	0.525 ± 0.028	< 0.0001
Hypertension	0.106 (0.040 to 0.170)	0.101 ± 0.032	0.002
Dyslipidaemia	− 0.095 (− 0.160 to − 0.029)	− 0.081 ± 0.028	0.005
Diabetes mellitus	− 0.030 (− 0.096 to 0.036)	− 0.028 ± 0.031	0.369
Men only—left common carotid artery IMT			
Age	0.472 (0.422 to 0.519)	0.477 ± 0.028	< 0.0001
Hypertension	− 0.001 (− 0.063 to 0.062)	− 0.001 ± 0.032	0.989
Dyslipidaemia	− 0.039 (− 0.101 to 0.023)	− 0.036 ± 0.029	0.212
Diabetes mellitus	0.077 (0.015 to 0.139)	0.076 ± 0.031	0.015
Men only—right common carotid artery IMT			
Age	0.488 (0.439 to 0.534)	0.492 ± 0.028	< 0.0001
Hypertension	0.074 (0.012 to 0.135)	0.074 ± 0.032	0.019
Dyslipidaemia	− 0.024 (− 0.086 to 0.038)	− 0.022 ± 0.028	0.444
Diabetes mellitus	0.001 (− 0.062 to 0.062)	0.001 ± 0.031	0.997
Young* only (n = 779)—left common carotid artery IMT			
Age	0.384 (0.322 to 0.442)	0.383 ± 0.033	< 0.0001
Gender (male)	0.199 (0.130 to 0.265)	0.185 ± 0.033	< 0.0001
Hypertension	− 0.001 (− 0.071 to 0.069)	− 0.001 ± 0.037	0.979
Dyslipidaemia	− 0.052 (− 0.122 to 0.018)	− 0.046 ± 0.032	0.146
Diabetes mellitus	0.084 (0.014 to 0.154)	0.086 ± 0.037	0.019
Young* only (n = 779)—right common carotid artery IMT			
Age	0.369 (0.306 to 0.428)	0.374 ± 0.034	< 0.0001
Gender (male)	0.128 (0.059 to 0.197)	0.121 ± 0.034	0.0003
Hypertension	0.064 (− 0.007 to 0.133)	0.068 ± 0.038	0.077
Dyslipidaemia	− 0.047 (− 0.117 to 0.023)	− 0.042 ± 0.032	0.190
Diabetes mellitus	0.035 (− 0.035 to 0.106)	0.037 ± 0.038	0.324
Old* only (n = 1109)—left common carotid artery IMT			
Age	0.291 (0.236 to 0.344)	0.288 ± 0.029	< 0.0001
Gender (male)	0.187 (0.129 to 0.243)	0.182 ± 0.029	< 0.0001
Hypertension	0.044 (− 0.015 to 0.102)	0.046 ± 0.031	0.148
Dyslipidaemia	− 0.057 (− 0.115 to 0.002)	− 0.055 ± 0.029	0.059
Diabetes mellitus	0.041 (− 0.019 to 0.099)	0.042 ± 0.031	0.178
Old* only (n = 1109)—right common carotid artery IMT			
Age	0.342 (0.288 to 0.393)	0.339 ± 0.028	< 0.0001
Gender (male)	0.159 (0.101 to 0.216)	0.151 ± 0.028	< 0.0001
Hypertension	0.101 (0.042 to 0.159)	0.104 ± 0.031	0.0008
Dyslipidaemia	− 0.053 (− 0.112 to 0.006)	− 0.051 ± 0.029	0.077
Diabetes mellitus	− 0.033 (− 0.092 to 0.026)	− 0.034 ± 0.030	0.269

*CI* confidence interval, *IMT* intima media thickness, *Std.* standardized; beta, slope. *Young, age < 50 years in men and age < 55 years in women; *Old, age ≥ 50 years in men and age ≥ 55 years in women

### Thresholds for increased IMT: need to assess both left and right sides

**Fig. 4:**
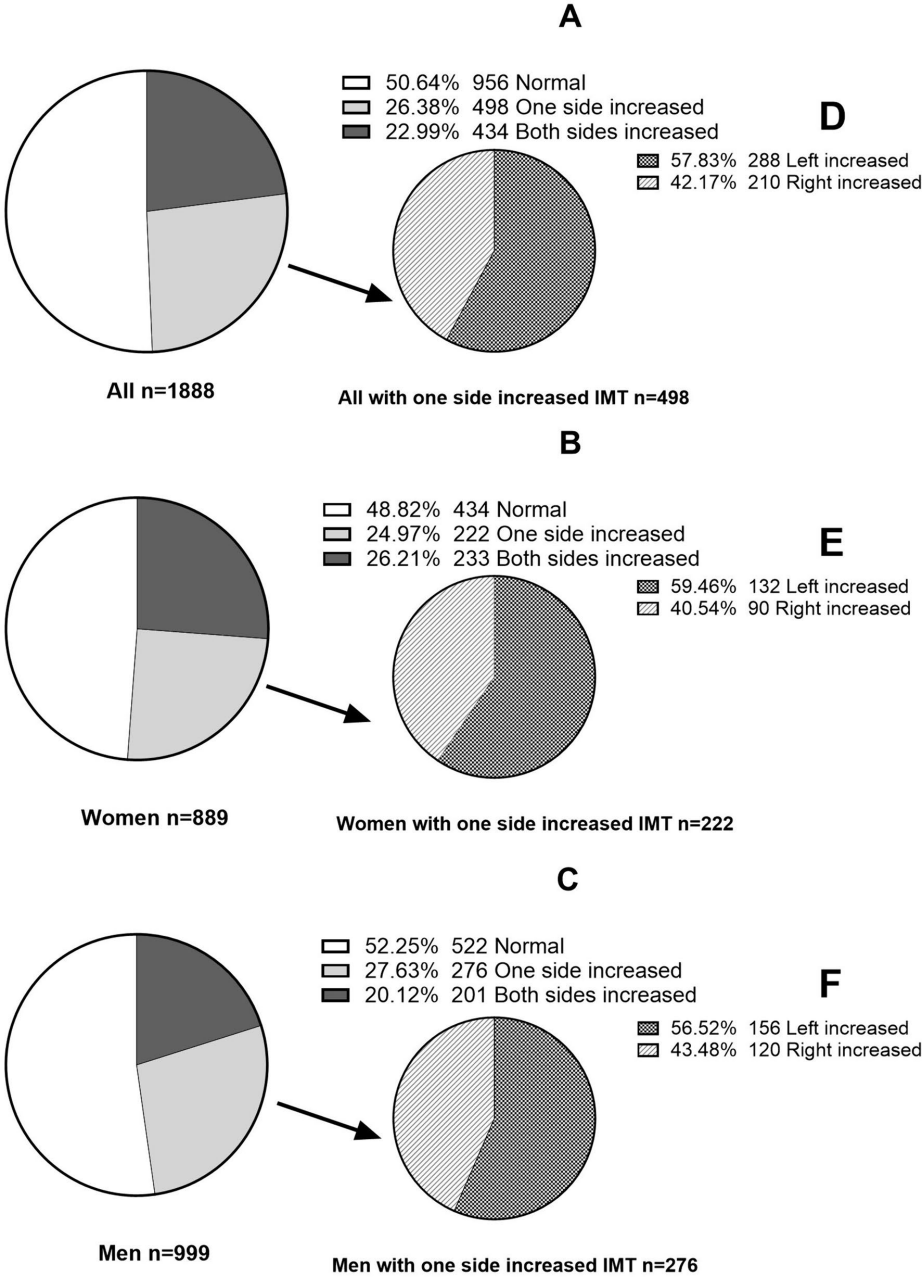
Proportions with increased common carotid IMT based upon age and gender specific thresholds [4], on one side or both sides in the study sample (**A**), and in women (**B**) and men (**C**) separately, and proportions with either increased left or right common carotid IMT in those with increased IMT on only one side, in the study sample (**D**), and in women (**E**) and men (**F**) separately

Figure 4 and Table 3 show the proportions of all patients, and women and men separately, with increased common carotid artery IMT on the left as compared to the right side based upon various thresholds. Using the single threshold of ≥ 0.68 mm [10] the proportion of all patients with increased left common carotid artery IMT (50.42%) was greater (p < 0.001) than the proportion with increased right common carotid artery IMT (44.70%). However, when side specific thresholds (either median or 75th percentile derived from normotensive, non-diabetic, and non-dyslipidaemic patients from the study sample [n = 439]) were employed, the proportions with increased left common carotid artery IMT were similar to the proportions with increased right common carotid artery IMT (Table 3). Moreover, when side specific thresholds for age and gender [4] were employed, the proportions with increased left common carotid artery IMT were similar to the proportions with increased right common carotid artery IMT in all patients, and in women and men separately (Table 3). Despite similar proportions with increased left and right common carotid artery IMT when using side specific thresholds for age and gender [4], at least a quarter of all patients only had increased common carotid artery IMT on one side (Fig. 4A). The proportion of patients with increased common carotid artery IMT on only one side was similar in women (Fig. 4B) and in men (Fig. 4C). In the patients with increased common carotid artery IMT on only one side, a greater proportion (p < 0.0001) had increased left common carotid artery IMT compared to increased right common carotid artery IMT (Fig. 4D–F). In the patients with increased common carotid artery IMT on only one side, the proportion with increased left common carotid artery IMT compared to increased right common carotid artery IMT was similar in women (Fig. 4E) and men (Fig. 4F).

**Table 3:** Proportions of patients with increased left or right common carotid artery IMT according to various thresholds in all patients and in women and men separately

Threshold	n	(%)
All patients (n = 1888)		
Left CCA IMT ≥ 0.713 mm (median)	790	41.84
Right CCA IMT ≥ 0.695 mm (median)	761	40.31
All patients (n = 1888)		
Left CCA IMT ≥ 0.84 mm (75th percentile)	354	18.75
Right CCA IMT ≥ 0.80 mm (75th percentile)	350	18.54
All patients (n = 1888)		
Left CCA IMT ≥ threshold (age, gender)*	722	38.24
Right CCA IMT ≥ threshold (age, gender)*	644	34.11
Women only (n = 889)		
Left CCA IMT ≥ 0.713 mm (median)	311	34.98
Right CCA IMT ≥ 0.695 mm (median)	320	36.00
Women only (n = 889)		
Left CCA IMT ≥ 0.84 mm (75th percentile)	125	14.06
Right CCA IMT ≥ 0.80 mm (75th percentile)	123	13.84
Women only (n = 889)		
Left CCA IMT ≥ threshold (age, gender)*	365	41.06
Right CCA IMT ≥ threshold (age, gender)*	323	36.33
Men only (n = 999)		
Left CCA IMT ≥ 0.713 mm (median)	479	47.95
Right CCA IMT ≥ 0.695 mm (median)	441	44.14
Men only (n = 999)		
Left CCA IMT ≥ 0.84 mm (75th percentile)	229	22.92
Right CCA IMT ≥ 0.80 mm (75th percentile)	227	22.72
Men only (n = 999)		
Left CCA IMT ≥ threshold (age, gender)*	357	35.74
Right CCA IMT ≥ threshold (age, gender)*	321	32.13

*CCA* common carotid artery, *IMT* intima media thickness

*Based upon age and gender specific thresholds as defined in Stein et al. [4]

### Determinants of IMT

On bivariate analyses, age, gender, hypertension, dyslipidaemia and diabetes mellitus were all strongly associated with left common carotid artery IMT (Table S1). Similarly, on bivariate analyses, age, gender, hypertension, and dyslipidaemia were all strongly associated with right common carotid artery IMT (Table S1). However, diabetes mellitus was only weakly associated with right common carotid artery IMT (Table S1). The relationships between common carotid artery IMT and its determinants were all positive, except for dyslipdaemia, which was inversely related to both left and right common carotid artery IMT (Table S1). However, the number of risk factors was not associated with either right or left carotid artery IMT (Table S1).

On multivariate analyses, age and gender were strongly associated with both left and right common carotid artery IMT (Table 2). However, left common carotid artery IMT was weakly associated with dyslipidaemia (inverse) and diabetes mellitus, but not associated with hypertension (Table 2). In comparison, right common carotid artery IMT was strongly associated with hypertension, weakly associated with dyslipidaemia (inverse), but not associated with diabetes mellitus (Table 2). Similarly, on multivariate analyses in women and men separately, age was strongly associated with both left and right common carotid artery IMT (Table 2). In women, left common carotid artery IMT was weakly associated with hypertension and dyslipidaemia (inverse), but not associated with diabetes mellitus (Table 2); whereas in men, left common carotid artery IMT was weakly associated with diabetes mellitus, but not associated with either hypertension or dyslipidaemia (Table 2). In both women and men, the right common carotid artery IMT was strongly associated hypertension, but not associated with diabetes mellitus (Table 2). In women, but not in men, the right common carotid artery IMT was weakly associated with dyslipidaemia (inverse). On multivariate analyses in young and old separately, the left common carotid artery IMT was associated with diabetes mellitus in the young, and weakly associated with dyslipidaemia (inverse) in the old (Table 2). In neither age group was the left common carotid artery IMT associated with hypertension (Table 2). In comparison, the right common carotid artery IMT was associated with hypertension (weakly in young, and strongly in old) (Table 2).

### Determinants of increased common carotid artery IMT

Tables 4, S2 and S3 show the determinants of increased common carotid artery IMT according to various thresholds in all patients (Table 4) and in women (Table S2) and men (Table S3) separately. In all patients, irrespective of the threshold employed, the main determinants of increased left or right common carotid artery IMT were age and gender (Table 4). In addition to age and gender, increased left common carotid artery IMT was determined by dyslipidaemia (inverse) and diabetes mellitus (Table 4). In comparison, in addition to age and gender, increased right common carotid artery IMT was determined by hypertension (Table 4). Similar to all patients, in both women and men separately, irrespective of the threshold employed, the main determinant of increased left or right common carotid artery IMT was age (Tables S2, S3). In women, in addition to age, increased left common carotid artery IMT was determined by dyslipidaemia, but only when using a threshold of above the median (Table S2); whereas, increased right common carotid artery IMT was determined by hypertension irrespective of the threshold employed (Table S2). In men, in addition to age, increased left common carotid artery IMT was determined by diabetes mellitus, and increased right common carotid artery IMT was determined by hypertension (Table S3).

**Table 4:** Determinants (per one SD) of increased left and right common carotid IMT in multivariate models in all patients (n = 1888)

Determinant (SD)	Odds ratio	(95% CI)	p value
Increased left CCA IMT (> 0.713 mm, median for healthy)			
Age (12.03)	2.958	(2.603 to 3.376)	< 0.0001
Gender (male) (0.50)	1.449	(1.304 to 1.616)	< 0.0001
Hypertension (0.38)	1.096	(0.979 to 1.228)	0.1111
Dyslipidaemia (0.44)	0.877	(0.790 to 0.975)	0.0145
Diabetes mellitus (0.30)	1.191	(1.064 to 1.334)	0.0024
Increased right CCA IMT (> 0.695 mm, median for healthy)			
Age (12.03)	3.152	(2.764 to 3.612)	< 0.0001
Gender (male) (0.50)	1.305	(1.174 to 1.453)	< 0.0001
Hypertension (0.38)	1.202	(1.073 to 1.348)	0.0016
Dyslipidaemia (0.44)	0.858	(0.772 to 0.954)	0.0046
Diabetes mellitus (0.30)	1.035	(0.923 to 1.159)	0.5558
Increased left CCA IMT (> 0.840 mm, 75th percentile for healthy)			
Age (12.03)	2.723	(2.345 to 3.180)	< 0.0001
Gender (male) (0.50)	1.449	(1.272 to 1.653)	< 0.0001
Hypertension (0.38)	1.015	(0.886 to 1.158)	0.8304
Dyslipidaemia (0.44)	0.889	(0.787 to 1.007)	0.0626
Diabetes mellitus (0.30)	1.178	(1.036 to 1.336)	0.0115
Increased right CCA IMT (> 0.800 mm, 75th percentile for healthy)			
Age (12.03)	2.752	(2.366 to 3.218)	< 0.0001
Gender (male) (0.50)	1.473	(1.292 to 1.684)	< 0.0001
Hypertension (0.38)	1.202	(1.054 to 1.366)	0.0054
Dyslipidaemia (0.44)	0.897	(0.792 to 1.017)	0.0878
Diabetes mellitus (0.30)	0.978	(0.854 to 1.114)	0.7387
Increased left CCA IMT (> age and gender specific thresholds)*			
Age (12.03)	1.245	(1.130 to 1.373)	< 0.0001
Gender (male) (0.50)	0.879	(0.798 to 0.966)	0.0079
Hypertension (0.38)	1.029	(0.924 to 1.145)	0.5961
Dyslipidaemia (0.44)	0.923	(0.839 to 1.017)	0.1045
Diabetes mellitus (0.30)	1.157	(1.043 to 1.284)	0.0060
Increased right CCA IMT (> age and gender specific thresholds)*			
Age (12.03)	1.175	(1.065 to 1.298)	0.0014
Gender (male) (0.50)	0.902	(0.818 to 0.994)	0.0372
Hypertension (0.38)	1.181	(1.060 to 1.314)	0.0024
Dyslipidaemia (0.44)	0.943	(0.855 to 1.041)	0.2412
Diabetes mellitus (0.30)	0.991	(0.889 to 1.102)	0.8678

*CCA* common carotid artery, *CI* confidence interval, *IMT* intima media thickness; *SD* standard deviation

*Based upon age and gender specific thresholds as defined in Stein et al. [4]

## Discussion

The main findings of the present study are as follows: In a large sample of Caucasian patients referred for cardiovascular risk assessment, we compared the left and the right common carotid artery IMT measurements, and assessed whether age, gender and cardiovascular risk factors have differential effects on the left versus the right common carotid artery IMT. We found that the left common carotid artery IMT was larger than the right, but not in the young (< 30 years) or the elderly (> 69 years), and that this side difference was less in women than in men. In addition, the determinants of the left common carotid artery IMT differed from those of the right. In addition to age and gender, the left common carotid IMT was associated with dyslipidaemia (protective) and diabetes mellitus, whereas the right common carotid artery IMT was correlated with hypertension. The apparent protective effect of dyslipdaemia was a reflection of the high prevalence of statin use (in accordance with current therapeutic practice, all patients diagnosed with dyslipidaemia were receiving lipid lowering medication [statins]).

The finding of a larger left compared to right common carotid artery IMT in the present study is consistent with previous reports of side differences in carotid IMT in various populations [8–16]. Moreover, our data support a former study indicating an impact of age on the presence of side differences in measurements of carotid artery IMT [9]. In this regard, our report of a greater left versus right carotid artery IMT in healthy individuals aged between 30 and 69 years, but no differences in those less than 30 or greater than 69 years of age is similar to that of Luo et al. [9] who reported a greater left versus right carotid artery IMT in healthy individuals aged between 35 and 65 years of age [9]. However, we extend these findings by reporting that gender, in addition to age, impacts on the side differences in carotid artery IMT. Similar to a previous report in a healthy Argentinean population [14], we report a greater difference in left versus right carotid IMT in men compared to in women, and that gender impacts on the effects of age on the differences between left and right carotid artery IMT.

Reasons for the impact of age on side differences in carotid artery IMT have been speculated. It has been suggested the side differences are not present in the young as it takes time for cardiovascular risk factors to have an impact on carotid artery IMT [13]. In addition, the increase in prevalence of cardiovascular risk factors with age may also play a role. However, this does not explain the lack of side differences in carotid artery IMT after the age of 65 years (present study) [9], and the presence of side differences in carotid artery IMT in a prior study in youth [23].

Reasons for the impact of gender on the effects of age on side differences in carotid artery IMT are unclear. However, in the present study, the men had a greater prevalence of hypertension and diabetes mellitus, and the left common carotid artery IMT was associated with diabetes mellitus in men but not in women. Indeed, the greater carotid artery IMT in men compared to in women [8, 24–26], has been attributed to a greater prevalence of cardiovascular risk factors in men. These data suggest that the reporting of measurements on both the left and the right common carotid artery IMT is particularly pertinent in men.

A theoretical suggestion for side differences in carotid artery IMT, is the dissimilarity in the anatomical origins of the carotid arteries from the aorta. In this regard, the left carotid artery comes directly off the aortic arch, whereas the right carotid artery originates from the innominate artery which is an extension of the ascending aorta. Consequently, blood flow velocity and oscillating shear forces, which are strong determinants of intimal thickening, are greater in the left compared to the right carotid artery [16]. In comparison, Luo et al. [9] showed that the right carotid artery IMT was associated with haemodynamic parameters (blood flow velocity); whereas the left carotid artery IMT correlated better with blood biochemical indices such as blood cholesterol (total and LDL-cholesterol) and blood glucose levels. We extend these findings by showing that independent of age and gender, the right common carotid artery IMT was associated with hypertension; while the left common carotid artery IMT was associated with diabetes mellitus and dyslipidaemia. Moreover, we show that the right common carotid IMT was consistently associated with hypertension irrespective of gender; whereas the left common carotid artery IMT was associated with diabetes mellitus in men and dyslipidaemia in women. The lack of association of the number of risk factors with either right or left carotid artery IMT, could be attributed to the apparent protective impact of dyslipidaemia on carotid artery IMT. In other words, the positive association of either hypertension with right or diabetes with left carotid artery IMT, is offset by the negative association of dyslipidaemia with carotid artery IMT. Although metabolic syndrome has been associated with carotid artery IMT in both normotensive and hypertensive participants [29], in meta-analyses [30], and in longitudinal studies [31, 32], whether metabolic syndrome is associated differentially with right versus left carotid IMT needs to be assessed in future studies.

There are several limitations to the present study. As the present study was cross-sectional in design, the relationships noted may not be cause and effect and may be attributed to residual confounding. Further studies evaluating the long-term impact of haemodynamic and biochemical cardiovascular risk factors on left versus right carotid artery IMT are therefore required. Second, we employed a combination of manual and semi-automated measurements of carotid artery IMT. However, it has previously been reported that the method of measurement of carotid artery IMT has no impact on the presence versus absence of side differences in carotid artery IMT [13, 15]. In addition, although automated echo-tracking is advantageous as it lowers the variability between readers [33], in the current study all of the IMT measurements were performed by the same investigator (BS). Third, data on lipid lowering and anti-hypertensive therapy were not available, and hence could not be included in the models. However, most patients were receiving lipid lowering therapy (74.5% had dyslipidaemia and were receiving lipid lowering agents as per current clinical practise). It is possible that therapy could be a determinant of IMT. Indeed, the negative relationship observed between dyslipidaemia and IMT is likely a reflection of the high prevalence of lipid lowering therapy. Bearing in mind the relationship between hypertension and right carotid artery IMT, and between diabetes and left carotid artery IMT in the current study, it is possible that anti-hypertensive therapy may influence the progression of right carotid artery IMT, and anti-diabetic agents may influence the progression of left carotid artery IMT. However, future studies should investigate the impact of current therapy on left versus right carotid artery IMT. Four, due to the low prevalence of data from other population groups, the present study was only conducted in Caucasian participants. Hence, whether the results are translatable to all populations is unknown. Nevertheless, our data are supported in part by similar findings in an Argentinean population [14], and in a Chinese population [9]. Lastly, the smaller sample sizes of individuals less than 30 years and older than 69 years of age, may in-part explain the lack of differences between left and right carotid artery IMT within these age groups. However, our findings support those previously reported in a Chinese population [9], where no side differences in IMT were observed in individuals less than 35 or more than 65 years of age, and the sample sizes for these groups were similar to the group aged 56 to 65 years where side differences were noted [9].

The presence of side differences in common carotid artery IMT and the differential effects of cardiovascular risk factors on the right compared to the left carotid artery IMT are of clinical relevance. Disparities in the prognostic ability of the right versus the left carotid artery IMT have previously been reported [21, 34–36]. In patients with coronary artery disease, the right carotid artery IMT (hazard ratio [HR] 18.31, p < 0.0001) predicted cardiovascular outcomes, but not the left carotid artery IMT (HR 3.81, p = 0.11). Furthermore, the predictive ability of the right carotid artery IMT was independent of confounders in multivariate analyses (HR 17.07, p = 0.007) [21]. As the patients had coronary artery disease, the cardiovascular outcomes were predominantly cardiac in origin (27% cardiac death, 73% acute coronary syndrome). In a community-based study of the elderly, a 0.3 mm increase in right carotid artery IMT was a better predictor of all-cause mortality (right IMT: relative risk [RR] = 3.33, p = 0.0053; left IMT: RR = 1.65, p = 0.022) and cardiovascular mortality (right IMT: RR = 2.89, p = 0.038; left IMT = 2.35, p = 0.043) [34], than the same increase in left carotid artery IMT. One third of the deaths in this study were cardiovascular, being either myocardial infarction or stroke. The superior predictive ability of the right carotid artery IMT over the left carotid artery IMT, may be attributed to its relationship with hypertension. In the current study, the right, but not the left, carotid artery IMT was associated with hypertension. In addition, both borderline hypertension and isolated systolic hypertension are associated with increases in the right, but not the left, carotid artery IMT [35, 36]. These data would suggest that the right carotid artery IMT is more sensitive to haemodynamic cardiovascular risk factors and consequently better predicts clinical events than the left carotid artery IMT. The differential power of the right and left carotid artery IMT in predicting vascular events, depending on the patients baseline characteristics and pathogenesis, is important to consider in the interests of personalised and precision medicine.

In conclusion, in a large sample of Caucasian patients referred for cardiovascular risk assessment, we show that age, gender and cardiovascular risk factors have differential effects on the left versus the right common carotid artery IMT. In this regard, the left common carotid artery IMT was larger than the right, but not in the young (< 30 years) or the elderly (> 69 years), and this side difference was less in women than in men. In addition to age and gender, the left common carotid IMT was associated with dyslipidaemia and diabetes mellitus, whereas the right common carotid artery IMT was associated with hypertension. These data suggest that there may be subgroups, such as men and those aged 30 to 69 years, where reporting on the left and right carotid artery IMT as opposed to the average is particularly pertinent. Moreover, in the present study, only 25% of participants had increased IMT on both the left and the right sides. As the left and right common carotid artery IMT may exhibit different prognostic values in patients with stable coronary artery disease [21], our data highlight the importance of assessing common carotid artery IMT on both sides of the body.

## Supplementary Information

Below is the link to the electronic supplementary material.

Supplementary file1 (DOCX 23 KB)

## Data Availability

The data to support the findings in this study are available from the researchers upon reasonable request.
