# Supplementary material for: Left versus right carotid artery IMT: differential impact of age, gender, and cardiovascular risk factors
Source: Int J Cardiovasc Imaging. 2024 Sep 26;40(11):2391–404. doi: 10.1007/s10554-024-03245-1 (PMC11561018; doi:10.1007/s10554-024-03245-1)
Supplement: Supplementary file 1 — Supplementary file1 (DOCX 23 KB) [file 10554_2024_3245_MOESM1_ESM.docx]

**On-line Supplement**

**Left versus right carotid artery IMT – Differential impact of age, gender, and cardiovascular risk factors.**

Belinda Stevens, Talib Abdool-Carrim, Angela J Woodiwiss.

From the Cardiovascular Pathophysiology and Genomics Research Unit, School of Physiology, Faculty of Health Sciences, University of the Witwatersrand, Johannesburg, South Africa.

Running title: Left vs right carotid IMT and risk factors.

BS and AJW contributed equally to this work.

Correspondence and reprint requests: Angela J Woodiwiss, Cardiovascular Pathophysiology and Genomics Research Unit, School of Physiology, University of the Witwatersrand Medical School, 7 York Road, Parktown, 2193, Johannesburg, South Africa. Tel + 27 11 717 2363, e-mail: [angela.woodiwiss@wits.ac.za](mailto:angela.woodiwiss@wits.ac.za)

**Table S1**. Individual determinants (bivariate models) of left common carotid artery IMT and right common carotid artery IMT.

Determinant Pearsons’s r (95% CI) p value

Left common carotid artery IMT

Age 0.473 (0.437 to 0.507) <0.0001

Gender (male) 0.163 (0.119 to 0.207) <0.0001

Hypertension 0.110 (0.065 to 0.155) <0.0001

Dyslipidaemia -0.101 (-0.145 to -0.056) <0.0001

Diabetes mellitus 0.085 (0.040 to 0.129) =0.0002

Number of risk factors 0.030 (-0.015 to 0.075) =0.198

Right common carotid artery IMT

Age 0.503 (0.469 to 0.536) <0.0001

Gender (male) 0.121 (0.077 to 0.166) <0.0001

Hypertension 0.137 (0.093 to 0.181) <0.0001

Dyslipidaemia -0.096 (-0.140 to -0.051) <0.0001

Diabetes mellitus 0.045 (0.001 to 0.090) =0.0497

Number of risk factors 0.031 (-0.015 to 0.076) =0.184

CI, confidence interval; IMT, intima media thickness.

**Table S2**. Determinants (per one SD) of increased left and right common carotid IMT in multivariate models in women only (n=889).

Determinant (SD) Odds Ratio (95% CI) p value

Increased left CCA IMT (>0.713 mm, median for healthy)

Age (12.31) 3.359 (2.730 to 4.187) <0.0001

Hypertension (0.34) 1.225 (1.041 to 1.444) =0.0148

Dyslipidaemia (0.42) 0.843 (0.722 to 0.985) =0.0314

Diabetes mellitus (0.23) 1.059 (0.898 to 1.245) =0.4934

Increased right CCA IMT (>0.695 mm, median for healthy)

Age (12.31) 3.070 (2.519 to 3.787) <0.0001

Hypertension (0.34) 1.173 (0.997 to 1.379) =0.0541

Dyslipidaemia (0.42) 0.807 (0.693 to 0.940) =0.0058

Diabetes mellitus (0.23) 1.017 (0.862 to 1.195) =0.8374

Increased left CCA IMT (>0.840 mm, 75^th^ percentile for healthy)

Age (12.31) 2.759 (2.153 to 3.588) <0.0001

Hypertension (0.34) 1.142 (0.932 to 1.385) =0.1870

Dyslipidaemia (0.42) 0.903 (0.746 to 1.102) =0.3058

Diabetes mellitus (0.23) 1.080 (0.878 to 1.307) =0.4467

Increased right CCA IMT (>0.800 mm, 75^th^ percentile for healthy)

Age (12.31) 2.811 (2.185 to 3.670) <0.0001

Hypertension (0.34) 1.223 (1.000 to 1.484) =0.0450

Dyslipidaemia (0.42) 0.797 (0.659 to 0.967) =0.0878

Diabetes mellitus (0.23) 0.993 (0.794 to 1.215) =0.7387

Increased left CCA IMT (>age and gender specific thresholds)*

Age (12.31) 1.300 (1.130 to 1.499) =0.0003

Hypertension (0.34) 1.069 (0.918 to 1.244) =0.3854

Dyslipidaemia (0.42) 0.935 (0.816 to 1.073) =0.3361

Diabetes mellitus (0.23) 1.090 (0.939 to 1.266) =0.2571

Increased right CCA IMT (>age and gender specific thresholds)*

Age (12.31) 1.224 (1.062 to 1.415) =0.0057

Hypertension (0.34) 1.152 (0.989 to 1.341) =0.0685

Dyslipidaemia (0.42) 0.929 (0.809 to 1.068) =0.2983

Diabetes mellitus (0.23) 1.026 (0.881 to 1.191) =0.7378

CCA, common carotid artery; CI, confidence interval; IMT, intima media thickness; SD, standard deviation. *based upon age and gender specific thresholds as defined in Stein et al (2008) [4].

**Table S3**. Determinants (per one SD) of increased left and right common carotid IMT in multivariate models in men only (n=999).

Determinant (SD) Odds Ratio (95% CI) p value

Increased left CCA IMT (>0.713 mm, median for healthy)

Age (11.74) 2.708 (2.308 to 3.200) <0.0001

Hypertension (0.41) 1.011 (0.865 to 1.181) =0.8892

Dyslipidaemia (0.45) 0.906 (0.786 to 1.045) =0.1754

Diabetes mellitus (0.35) 1.288 (1.105 to 1.505) =0.0013

Increased right CCA IMT (>0.695 mm, median for healthy)

Age (11.74) 3.196 (2.692 to 3.826) <0.0001

Hypertension (0.41) 1.221 (1.042 to 1.434) =0.0138

Dyslipidaemia (0.45) 0.905 (0.781 to 1.048) =0.1807

Diabetes mellitus (0.35) 1.044 (0.892 to 1.221) =0.5900

Increased left CCA IMT (>0.840 mm, 75^th^ percentile for healthy)

Age (11.74) 2.679 (2.230 to 3.247) <0.0001

Hypertension (0.41) 0.941 (0.783 to 1.124) =0.5069

Dyslipidaemia (0.45) 0.884 (0.753 to 1.039) =0.1326

Diabetes mellitus (0.35) 1.249 (1.054 to 1.478) =0.0098

Increased right CCA IMT (>0.800 mm, 75^th^ percentile for healthy)

Age (11.74) 2.704 (2.248 to 3.282) <0.0001

Hypertension (0.41) 1.183 (0.994 to 1.405) =0.0565

Dyslipidaemia (0.45) 0.971 (0.824 to 1.147) =0.7237

Diabetes mellitus (0.35) 0.969 (0.810 to 1.153) =0.7297

Increased left CCA IMT (>age and gender specific thresholds)*

Age (11.74) 1.192 (1.043 to 1.365) =0.0104

Hypertension (0.41) 1.001 (0.860 to 1.162) =0.9919

Dyslipidaemia (0.45) 0.912 (0.797 to 1.044) =0.1795

Diabetes mellitus (0.35) 1.206 (1.046 to 1.392) =0.0101

Increased right CCA IMT (>age and gender specific thresholds)*

Age (11.74) 1.131 (0.986 to 1.298) =0.0787

Hypertension (0.41) 1.200 (1.032 to 1.394) =0.0172

Dyslipidaemia (0.45) 0.953 (0.830 to 1.096) =0.4976

Diabetes mellitus (0.35) 0.969 (0.883 to 1.122) =0.6774

CCA, common carotid artery; CI, confidence interval; IMT, intima media thickness; SD, standard deviation. *based upon age and gender specific thresholds as defined in Stein et al (2008) [4].
